# Nutrient Stability in NASA Spaceflight Experiment Rodent Food Bars

**DOI:** 10.3390/foods13244093

**Published:** 2024-12-18

**Authors:** Janani Iyer, Tyler S Marsh, Ryan J Fisher, Vandana Verma

**Affiliations:** 1 Universities Space Research Association, Mountain View, CA 94043, USA; 2 KBR, Houston, TX 77002, USA; 3 Space Biosciences, NASA Ames Research Center, Moffett Field, CA 94035, USA; 4 ASRC Federal Space and Defense, 7000 Muirkirk Meadows Drive, Beltsville, MD 20705, USA

**Keywords:** Rodent, spaceflight, food bars, nutrients

## Abstract

The Nutrient-upgraded Rodent Food Bar (NuRFB) is the standard diet for mice in NASA’s Rodent Research Project aboard the International Space Station (ISS). Given the nature of spaceflight and the lengthy production process of the food bars, a shelf-life assessment was conducted to evaluate nutritional stability over time (ranging from 0 to 27 months) and under different storage conditions (refrigerated, ambient, and refrigerated + ambient), where ambient is 22–23 °C. Lipid oxidation markers and fat- and water-soluble vitamins were assessed under various time and temperature conditions using AOAC International methods. Vitamin D levels showed a minor decrease, and riboflavin fluctuated slightly over time, but all vitamin levels remained above National Research Council (NRC) minimum requirements. Food bars stored at 4 °C showed significantly higher thiamine levels than the bars that underwent some degree of ambient temperature storage, but all met the NRC guidelines. Minimal lipid oxidation was observed for up to 18 months, and no mold or yeast growth occurred despite the high moisture content of the bars. This study confirms that NuRFBs maintain stable vitamin and lipid oxidation indices, ensuring adequate nutrition for rodents during spaceflight.

## 1. Introduction

The spaceflight environment presents several unique challenges, including microgravity, ionizing radiation, isolation, altered circadian rhythm, and elevated CO_2_ levels, among other factors. These factors pose significant risks to various tissues and thus affect human health in space [1,2]. Rodent spaceflight research has continually provided insights into our understanding of the physiological effects of spaceflight on musculoskeletal, immune, cardiovascular, sensorimotor, and nervous systems [3,4]. As rodent research paves the way to strengthen astronaut health during long-duration deep space missions, the rodent diet provided during these experiments is an important aspect to consider as it affects essentially all physiological systems [5,6]. Several studies have demonstrated that gut microbiota in rodent research plays an integral role in rodent phenotype, experimental reproducibility, and translatability of data obtained [7,8,9].

In terrestrial research, the American Institute of Nutrition established the purified-ingredient AIN-76A diet followed by a reformulated AIN-93 diet to enable researchers across the globe to meet the nutritional needs of rodents [10,11]. A nutritionally rich and stable rodent diet is an essential aspect of these studies, especially considering the limitations and the challenges posed by the nature of spaceflight studies. In lieu of the unique spaceflight environment altering the nutritional requirements of rodents, NASA’s Rodent Research Project developed the Nutrient-upgraded Rodent Food Bar (NuRFB) [5,12]. The semi-purified NuRFB is the current standard diet for mice involved in experiments in spaceflight missions. It is based on the AIN-93G diet with certain additions to meet nutrient requirements for rodents. In addition, the texture of the food bar is an essential consideration in the microgravity environment to prevent the crumbling of the bar for sanitary and safety reasons [5,12]. The NuRFBs have been used successfully in several rodent Space Shuttle and International Space Station (ISS)-based missions. As NASA prepares for long-duration missions beyond low Earth orbit to the Moon and Mars, extending the shelf life of the NuRFBs is necessary. The purpose of this study was to assess the nutrient stability of the NuRFB at ambient and refrigerated storage conditions over increasing storage time.

The exiguous supply of cold stowage on the ISS necessitated further exploration as to whether NuRFB can be stored at ambient temperatures (AT) without excessive nutrient degradation. The oxidative and nutritive stability of NuRFBs has previously been tested over a one-year period in order to evaluate storage effects at controlled temperatures of either 4 °C (refrigerated) or 26 °C (ambient) [12]. However, these tests were continuously sampled from only one food bar sample that was repeatedly exposed to ambient air in an uncontrolled environment. NuRFBs contain 5% fat from soybean oil, which has increased fatty acids that are prone to degradation via oxidation [12]. Exposure to uncontrolled ambient air, which is 20.95% oxygen, increases the rate of oxidation of fatty acids exposed to this environment. Further, excessive oxidation leads to rancidity, which in turn affects the palatability of the food. Continued degradation may lead to toxicity and nutritive deficiency in the food when consumed by rodents as the sole source of nutrition. Additionally, auto-oxidation of polyunsaturated fatty acids (PUFAs) of edible vegetable oils results in the formation of fatty acid hydroperoxides that can undergo further chemical transformations to yield a variety of re-arranged and side-chain cleavage products. Since the oxidation products of PUFAs have been shown to have cytotoxic and pro-inflammatory effects [13], the consumption of rancid oils may have unforeseen confounding effects on the outcomes of the research objectives. Thus, for both animal welfare and scientific reasons, it is vital to provide shelf-stable, palatable, and fresh food bars to rodents.

The main objective of this study was to evaluate the stability of nutrients in the NuRFBs in different storage conditions over an extended period of time. In this study, we systematically analyzed the food bars at multiple time points (ranging from 0 to 27 months) and under different storage conditions (refrigerated, ambient, and refrigerated + ambient), where ambient is 22–23 °C (as maintained on ISS). A panel of lipid oxidation markers and both fat- and water-soluble vitamins were assessed under various time and temperature conditions via the Association of Official Analytical Collaboration (AOAC) International standardized methodology. The food bars were also assessed for mold and microbial growth over an extended period. Overall, no meaningful nutritional changes were observed, and all nutrients analyzed were well within the National Research Council guidelines for rodents. Our results do not suggest a time or temperature-dependent loss of nutrient stability or increase in lipid oxidation indices, and while refrigeration can provide a slight increase in the shelf-life, the nutrient profile of the food bars stored at ambient temperature was not severely affected under sterile and unopened conditions.

## 2. Methods

### 2.1. NuRFB Composition

NuRFB is an iteration of the original NASA Rodent food bar, wherein the formulation was modified to minimize vitamin degradation during the production process. The formulation is outlined in Table 1. The NuRFB production involves feeding the dry diet powder into a twin-screw extrusion machine where water is added, and the mix is heated, cooked, and extruded using a custom metal die before trimming the bar to meet the necessary volumetric guidelines (1 inch × 8 inch × 1.25 inch). These bars are then dipped in a 15% potassium sorbate solution, dried, vacuum packaged, and sterilized via gamma irradiation (Cobalt-60; 15–25 kGy). A randomized representative selection of sterilized bars was analyzed to assess nutrient levels, and all the bars were stored at 4 °C until use.

### 2.2. NuRFB Sampling for Biochemical Analysis

**Figure 1:**
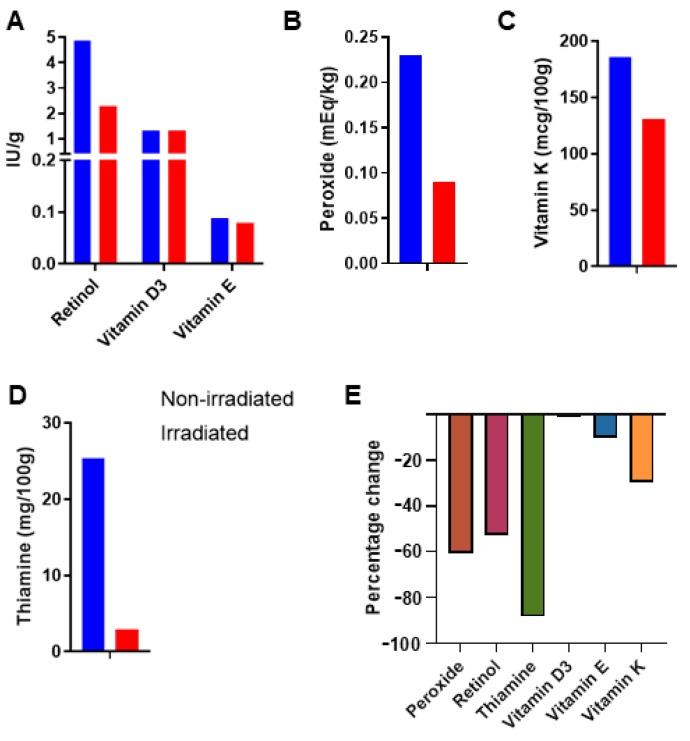
Nutrient and lipid oxidation analysis of NuRFBs in irradiated (red) and non-irradiated (blue) samples. Histogram represents fat-soluble vitamins retinol, D3, and E (**A**); peroxide (**B**); vitamin K1 (**C**); and thiamine (**D**) levels in the NuRFBs. (**E**) Bar graph showing the % loss of nutrients in irradiated NuRFBs. n = 1 for all tested nutrients.

**Figure 2:**
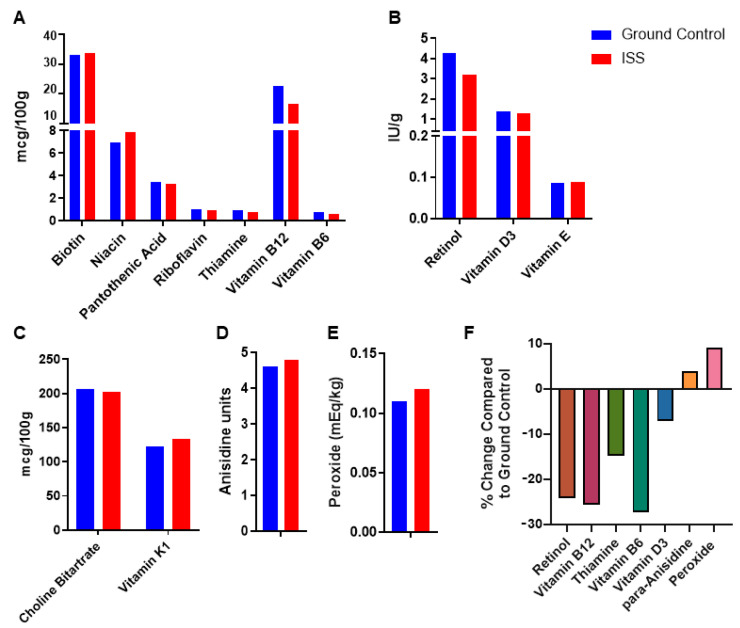
Rodent Research 5 (RR-5) mission NuRFB nutrient analysis. Histogram represents water-soluble vitamins (**A**); fat-soluble vitamins retinol and vitamins D3 and E (**B**); choline bitartrate and vitamin K1 (**C**); p-anisidine (**D**); and peroxide (**E**) levels in the NuRFBs. (**F**) Bar graph showing the % loss of nutrients in NuRFBs sent to the ISS compared to the ground controls. n = 1 for all tested nutrients.

NuRFBs are used in Flight and Ground control experiments within the Rodent Research Hardware System. Since spaceflight studies require irradiation sterilization of food prior to launch, samples (n = 1) were taken from both irradiated and non-irradiated conditions to assess the nutritional impact of irradiation (non-irradiated and irradiated samples referred to in Figure 1). Additionally, NuRFB samples (n = 1) were collected from bars sent to the ISS during the RR5 mission, along with their corresponding ground controls, to replicate flight conditions for nutrient analysis. These are referred to as ISS and Ground Control in Figure 2. The NuRFBs were transported to the ISS on the SpaceX Commercial Resupply Service (CRS)-11 and kept aboard the ISS for 15 weeks. For comparison, the ground control NuRFBs were stored at Kennedy Space Center (KSC) at 22–23 °C (similar to what is observed on the ISS) for the same duration prior to testing.

To determine the nutritional stability and shelf life of NuRFBs over extended periods under different storage conditions, sampling was conducted in different groups, as listed in Table 2. The experimental and control groups were designed to replicate real-world flight mission scenarios and test the limits of the current storage protocols. For each group, three samples (n = 3) were collected at the specified time points (Table 2), with each sample consisting of three food bars vacuum packed and prepared identically before nutrient analysis. The food bars were vacuum-sealed in Tyvek packaging to mimic flight conditions and not exposed to light during storage. All sample groups, except Group 1A, were packaged with a nitrogen purging step followed by vacuum sealing. Group 1A, instead of nitrogen purging, underwent ethylene oxide gas sterilization of the packaging (not the food) before vacuum sealing.

### 2.3. Nutrient and Lipid Oxidation Analysis

Samples were sent for analysis at the end of the assigned ambient and refrigerated storage period to Eurofins Food Integrity and Innovation, where they were refrigerated at 4 °C until analysis. The samples were assessed for lipid oxidation and nutrient stability using a panel of lipid oxidation markers, fat- and water-soluble vitamins, and microbiological assessments under various time and temperature conditions via AOAC International standard methodology (Table 3). Ambient temperature (AT) was defined as 22–23 °C, which aligns with the ambient temperature maintained on the International Space Station.

For the analysis of fat-soluble vitamins (vitamin A, vitamin D3, and vitamin E), the samples were first saponified to digest any lipids and release the vitamins. In the case of vitamins A and E, the saponification step was followed by extraction with an organic solvent. These vitamins were directly quantified as all-trans-retinol and 13-cis-retinol (vitamin A) [14,15] and alpha-tocopherol (vitamin E) by HPLC [16,17,18]. Meanwhile, vitamin D3 was extracted post-saponification via liquid/liquid partitioning, followed by drying, reconstitution, and analysis by liquid chromatography–tandem mass spectrometry (LC/MS/MS) [19].

The water-soluble vitamins were analyzed as described below:

(a) Riboflavin (microbiological method): The sample was first hydrolyzed with dilute hydrochloric acid and adjusted for pH. The amount of riboflavin was determined turbidimetrically by comparing the growth response of the sample using the bacteria *Lactobacillus rhamnosus* with the growth response of the riboflavin standard [20,21].
(b) Thiamine (fluorometric method): The sample was autoclaved under weakly acidic conditions followed by incubation with a buffered enzyme solution in order to complete the release of any bound thiamine. Further, this solution was purified on a cation exchange column. Potassium ferricyanide was added to an aliquot of the purified solution to convert thiamine to thiochrome, which was then extracted, read on a fluorometer, and quantified using an external standard [12,22,23,24].

In an oxygenated environment, fatty acids are prone to oxidation. Peroxide, a primary oxidation product, was measured as milli-equivalents of peroxide per kilogram of the sample [25,26]. Secondary oxidation products were assessed by measuring p-anisidine values [27,28]. Specifically, lipids were extracted from the samples, and the aldehydic compounds in the sample reacted with p-anisidine and acetic acid solution and measured at an absorbance of 350 nm. Additionally, the total oxidation value (TOTOX) was calculated using the peroxide value and p-anisidine value (TOTOX = p-anisidine value + (2 × peroxide value)).

Moisture content was determined by drying the sample in a vacuum oven at 100 ± 5 °C for 5 to 5.5 h [29,30]. Mold, yeast, and aerobic [31,32] and anaerobic bacteria CFUs were determined using standard microbiological plate count methods.

### 2.4. Statistical Analysis

Groups were compared with each other using Student’s *t*-tests (non-parametric Mann–Whitney) and one-way ANOVA (non-parametric Kruskal–Wallis) to test for significance. Data analysis and visualization were performed using GraphPad Prism. *p*-values ≤ 0.05 were considered significant.

## 3. Results

NuRFBs are the standard rodent diet in spaceflight experiments and have been carefully formulated to meet the nutritional requirements of laboratory rodents. Previous studies have shown nutrient losses during the manufacturing and processing of the NuRFBs, especially in the extrusion step [5]. The irradiation step (15–25 kGy C60) carried out post-extrusion also leads to a loss in nutrients, including decreases in vitamin A (53%), thiamine (89%), vitamin E (10%), vitamin D (2%), and vitamin K1 (30%) (Figure 1). These observations have been historically noted as the diet has been reformulated to account for these losses [5]. Although the small sample size in this study (n = 1) limits the ability to draw definitive conclusions, these preliminary findings offer valuable insights into the nutritional stability of the food bars after exposure to radiation.

The food bars are monitored routinely to assess their nutrient levels, and the rodent diet is supplemented accordingly to ensure there are no deficiencies. As flight missions using rodents increase in their duration, it is important to assess the effects of the spaceflight stressors, specifically galactic cosmic radiation (GCR), on the nutritional content of the food bars. The food bar from the RR5 spaceflight mission (a 15 week spaceflight mission on ISS) were subjected to nutrient analysis and tested for a panel of lipid oxidation markers and fat- and water-soluble vitamins. The two groups tested were the bar sent to the ISS and the ground control bar that remained in the Ground Flight Facility with no low earth orbit (LEO) component. The ISS bars showed decreases in retinol (24%), vitamin B12 (25%), thiamine (15%), vitamin B6 (28%), and vitamin D3 (7%) levels (Figure 2) compared to the ground controls. Meanwhile, the lipid oxidation markers in the ISS bars showed a slight increase, with para-anisidine at 4% and peroxide at 9%. Although there were changes noted in the ISS bars compared to the ground controls, they were well within the NRC requirements. A limitation of this study is the use of a sample size of n = 1 for both the spaceflight and ground control food bars, which was dictated by the constraints of the space mission, including limited sample space and weight restrictions. While this small sample size restricts the ability to draw definitive conclusions, the findings from these preliminary analyses still provide valuable insights into the nutritional stability of the food bars. Future studies will incorporate larger sample sizes to strengthen the reliability of the results.

**Figure 3:**
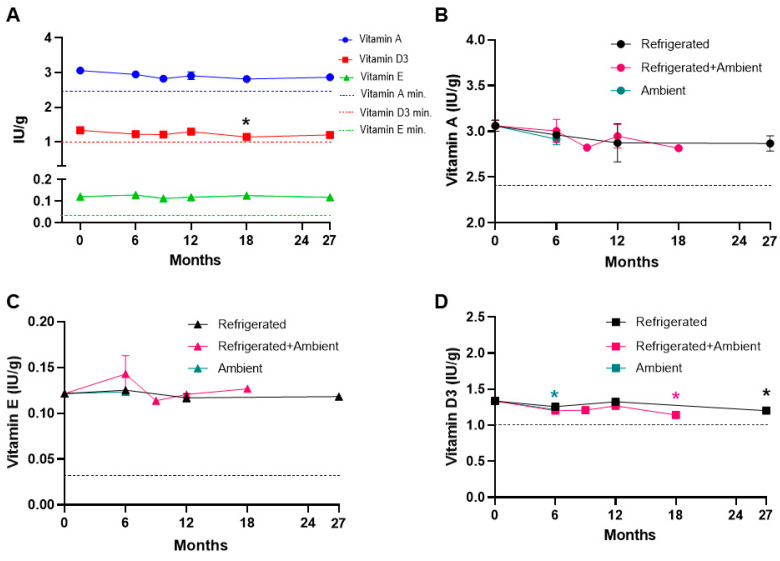
Fat-soluble vitamin levels in NuRFBs. (**A**) Vitamin A and E levels did not show any significant changes over 27 months in comparison to the baseline at 0 months, while a subtle yet significant loss of vitamin D3 levels was observed at the 18 (*p* = 0.0047, black *) time points. No significant changes were observed in the levels of vitamin A (**B**) and E (**C**) in the refrigerated, ambient, and refrigerated + ambient storage conditions compared to the baseline values. Vitamin D3 (**D**) levels were decreased in ambient storage at 6 months (*p* = 0.0119, teal *), and in refrigerated + ambient storage at the 18-month (*p* = 0.0134, pink *), and refrigerated storage at the 27-month (*p* = 0.037, black *) time points compared to the baseline. The dotted line corresponds to NRC minimum requirements.

The main objective of this study was to evaluate the nutrient stability and lipid oxidation over an extended time across different storage conditions (Table 2). We assessed the stability of fat-soluble vitamins A, D, and E in the NuRFBs. Over a period of 27 months, we did not see any significant changes in the stability of vitamins A and E (Figure 3A). The values shown in Figure 3A represent nutrient stability over different time points, irrespective of the storage conditions. These data were included to assess the overall stability of the nutrients over time, without specific consideration of the different storage conditions. We observed slight but non-significant fluctuations in vitamin A levels across the different groups, possibly due to its labile nature and difficulty extracting retinol, but vitamin A levels exceeded the NRC minimum requirement in all the groups (Figure S1). Overall, no significant changes were noted in the levels of vitamin A and E in the refrigerated, ambient, and refrigerated + ambient storage conditions compared to the baseline value, and their levels exceeded the NRC recommendations (Figure 3B,C). We noted a subtle yet significant decrease in vitamin D levels at the 18-month time point (*p* = 0.0047) (Figure 3A). Also, vitamin D levels were decreased in the ambient storage at 6 months (*p* = 0.0119) and in refrigerated + ambient storage at 18 months (*p* = 0.0134) compared to the baseline (Figure 3D). We also observed a significant decrease in vitamin D3 levels in group 5B (12 months refrigeration followed by 6 months at ambient temperature) compared to the baseline (Figure S1C). Despite the reduction in the vitamin D levels at certain time points, their levels were over the NRC minimum across various conditions at all time points (Figure 3A,D and Figure S1B). Another notable observation is that at the 6-month time point, there were no changes in the nutrient levels of vitamins A, E, and D at ambient and refrigerated + ambient storage conditions compared to the refrigerated storage. Similarly, there were no changes at the 12-month time point between refrigerated and refrigerated + ambient storage conditions. These results suggest that the food bars maintain the nutrient levels for up to 18 months at the different storage conditions tested in this study.

**Figure 4:**
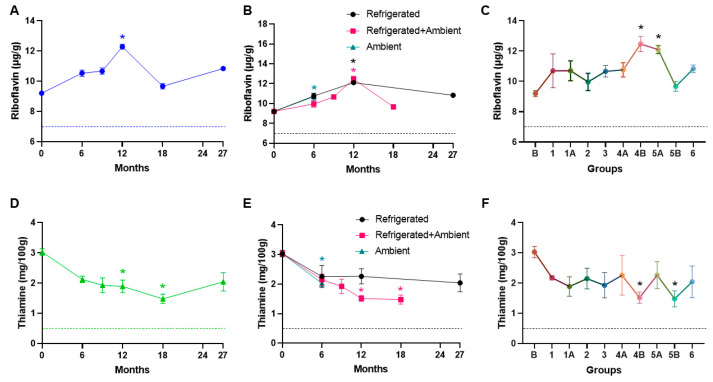
Water-soluble vitamin levels across time and different treatment groups. (**A**) An increasing trend was noted in riboflavin with a significant increase at the 12-month time point (*p* = 0.003, blue *). (**B**) Riboflavin levels were significantly increased at the 6-month time point in ambient (*p* = 0.0238, teal *) and at the 12-month point in refrigerated (*p* = 0.0066, black *) and refrigerated + ambient (*p* = 0.0055, pink *) storage conditions. (**C**) Different groups exhibited increasing levels of riboflavin with significant increases in groups 4B (6 months refrigeration followed by 6 months at ambient temperature, *p* = 0.0026, black *) and 5A (12-month refrigeration, *p* = 0.0063, black *) compared to the baseline-B. (**D**) A decreasing trend was noted in thiamine levels with significant decreases at 12-month (*p* = 0.047, green *) and 18-month (0.0058, green *) time points. (**E**) Thiamine levels were significantly decreased in ambient storage at the 6-month time point (*p* = 0.0238, teal *), as well as in refrigerated + ambient storage at 12 months (*p* = 0.0245, pink *) and 18 months (*p* = 0.0245, pink *) time points. (**F**) Different groups exhibited decreasing trends in thiamine levels with significant decreases in groups 4B (6 months refrigeration followed by 6 months at ambient temperature, *p* = 0.0089, black *) and 5B (12 months refrigeration followed by 6 months at ambient temperature, *p* = 0.0105, black *) compared to the baseline. The dotted line corresponds to the NRC minimum requirements for the respective vitamins.

We also assessed water-soluble vitamin levels, specifically riboflavin and thiamine. Over the course of 27 months and across different storage conditions, we observed an increasing trend in riboflavin level with a significant increase at the 12-month time point (*p* = 0.003) compared to the baseline value, possibly due to variability within the samples (Figure 4A–C). Figure 4A,D represents riboflavin and thiamine data over time, irrespective of their storage conditions. We also observed increased riboflavin levels at the 6-month time point in ambient (*p* = 0.0238) and at the 12 month point in refrigerated (*p* = 0.0066) and refrigerated + ambient (*p* = 0.0055) storage conditions. The increase in riboflavin observed during storage could be attributed to analytical variability inherent in the measurement process. Variations in sample preparation or instrument calibration, or even slight differences in the precision of nutrient analysis, could cause fluctuations in riboflavin levels. It is important to note that such variations are not uncommon, especially with micronutrient analysis, which can be sensitive to minor changes in technique or sample handling. It is important to note, however, that even with this observed variability, the riboflavin content in the samples remains above the NRC minimum requirement. This ensures that the food bars continue to meet the necessary nutritional standards for riboflavin, providing adequate levels for intended use. Overall, the riboflavin levels tested across time and different storage conditions exceeded the NRC recommendations, and no changes in were observed in food bars stored at ambient and refrigerated + ambient conditions compared to the refrigerated storage.

Thiamine is particularly susceptible to degradation during the extrusion process, and previous studies have reported up to a 21% loss [33]; hence, thiamine is supplemented in the dry diet to ensure there are no deficiencies in the diet that could lead to rodents not receiving adequate nutrition. In our assessment of thiamine levels in the food bars, we observed degradation over time with significant decreases at 12-month (*p* = 0.047) and 18-month (0.0058) time points (Figure 4D). We also noted a significant decrease in thiamine levels in food bars stored ambient conditions at the 6-month time point (*p* = 0.0238), as well as in refrigerated + ambient conditions at 12-month (*p* = 0.0245) and 18 months (*p* = 0.0245) time points (Figure 4E,F), suggesting that refrigeration might help with maintaining thiamine levels. Despite the observed decline in thiamine levels, they exceeded the NRC nutrient recommendations in all tested conditions.

**Figure 5:**
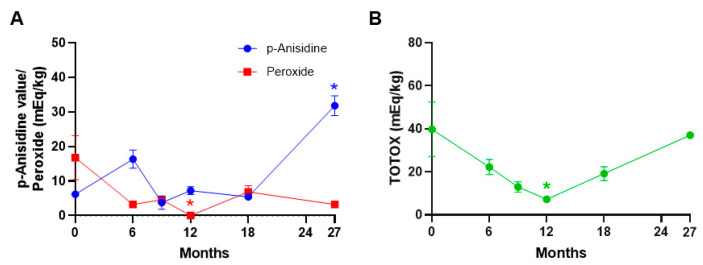
Lipid oxidation in NuRFBs. (**A**) Non-significant fluctuations in p-anisidine values were noted except at 27 months (*p* = 0.05) compared to baseline at 0 months, whereas the peroxide levels showed a decreasing trend with a significant decrease at 12 months (*p* = 0.0003). Red * represents statistical significance for peroxide levels, and blue * represents statistical significance for p-anisidine levels. (**B**) Reduction in total oxidation levels was observed over time with a significant decrease noted at the 12-month (*p* = 0.0079, green *) time point. * *p* ≤ 0.05.

To assess the oxidation and rancidity of the food bars, we measured the levels of peroxide and p-anisidine in the food bars. The peroxide levels are a measure of primary oxidation, and human food samples with levels between 10 and 20 mEq/kg are considered fresh and palatable [34]. There are, however, currently no such limits for rodent foods. Interestingly, we observed decreases in peroxide levels over time across different storage conditions compared to the baseline (Figure 5A and Figure S2A,D) with the peroxide levels below 10 mEq/kg, suggesting that the food bars’ oxidative status is not compromised even in ambient and refrigerated + ambient storage conditions. The secondary oxidation product, p-anisidine, showed an increasing trend at the 6-month time point and was significantly increased at the 27-month (*p* = 0.05) time point in the samples compared to the baseline (Figure 5A). While no significant changes were noted in p-anisidine levels in the ambient and refrigerated + ambient samples, refrigerated samples showed a significant increase at the 27-month time point compared to the baseline (*p* = 0.05), suggesting increased secondary oxidation during long-term storage (Figure S2E) and would be in line with typical kinetics of lipid oxidation. Although low peroxide levels were noted at 27 months, the high p-anisidine value at this time point suggests that the shelf life of these food bars may therefore be less than 27 months, despite refrigeration. Further, we observed a decreasing trend in TOTOX values over time, and a significant decrease was noted at 12 months (*p* = 0.0079) compared to the baseline (Figure 5B). While no significant changes were noted in TOTOX values in refrigerated samples, a significant decrease was noted in ambient storage at 6 months (*p* = 0.0476) and in refrigerated + ambient storage at the 12-month point (*p* = 0.0423) (Figure S2F). Overall, the peroxide levels, p-anisidine values, and TOTOX values were not significantly different in food bars stored at ambient and refrigerated + ambient conditions in comparison to refrigerated food bars, thus suggesting that the storage conditions do not affect the lipid oxidation in the time period tested.

**Figure 6:**
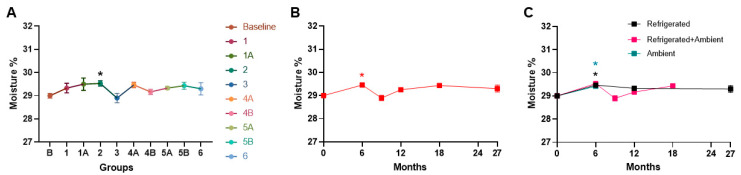
Moisture levels in NuRFBs. (**A**) Subtle fluctuations in moisture content were seen across groups with a significant increase in group 2 (3 months refrigeration followed by 3 months in ambient storage, *p* = 0.0427). Statistical significance for moisture is indicated by * (**B**) Similar fluctuations were observed in moisture levels over time with a significant increase at the 6-month time point (*p* = 0.0142), denoted by the red *. (**C**) Moisture levels were significantly increased at the 6-month time point in ambient (teal *, *p* = 0.0476), and refrigerated (black *, *p* = 0.0251) storage conditions compared to baseline.

**Figure 7:**
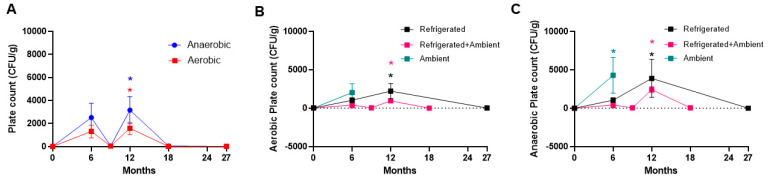
Microbial plate counts in NuRFBs. (**A**) Significant increase in aerobic (*p* = 0.0235) and anaerobic (*p* = 0.0107) plate counts were noted at 12 months compared to baseline at 0 months. Red * represents statistical significance for aerobic plate count, and blue * represents statistical significance for anaerobic plate count. (**B**) Aerobic plate counts were significantly increased at the 12-month point in refrigerated (*p* = 0.0437, black *) and refrigerated + ambient (*p* = 0.0440, pink *) storage conditions. (**C**) Anaerobic plate counts were significantly increased at the 6-month time point in ambient (*p* = 0.0238, teal *) and at the 12-month point in refrigerated (*p* = 0.0397, black *) and refrigerated + ambient (*p* = 0.0156, pink *) storage conditions.

Assessment of the moisture content of the NuRFBs at various storage conditions showed a slight increase in group 2 (3-month refrigeration followed by 3 months at ambient storage, *p* = 0.0427) (Figure 6A), at the 6-month time point (*p* = 0.0142) when assessed over time (Figure 6B), and at the 6-month time point in ambient (*p* = 0.0476) and refrigerated (*p* = 0.0251) storage conditions compared to the baseline (Figure 6). But overall, there were no major differences in moisture levels (Figure 6), which was likely due to the food bars being vacuum-sealed until use. During the microbiological testing of the NuRFBs, no significant differences were noted in mold and yeast counts from baseline values (Figure S3). While the aerobic and anaerobic plate counts showed some increase in samples at the 6- and 12-month time points compared to the baseline, there were no significant differences across the three storage conditions (Figure 7). This observed increase in some samples was probably due to variations in aseptic handling techniques, which have since been revised. There were no significant changes in aerobic and anaerobic plate counts across the different treatment groups (Figure S4).

## 4. Discussion

The spaceflight environment presents unique risks to humans, and rodent studies are instrumental in understanding how this environment impacts mammalian physiology. A nutritionally rich and stable rodent diet is an essential aspect of these studies, especially considering the limitations and the challenges of long term storage of food products posed by the nature of spaceflight studies [35]. Therefore, careful formulation of the NuRFBs must address and complement these unique demands. Few studies have documented the stability of the NuRFBs, with the last published study being over a decade ago. Since then, the NuRFB formulation has undergone multiple revisions to ensure it meets NRC requirements when presented to animals [10]. These revisions have primarily been driven by the necessity to irradiate the food for sterilization before spaceflight, as well as to account for nutrient losses incurred during extrusion [36,37]. With a moisture content of 30%, food bars are also vacuum-sealed to prevent mold formation. It has been historically noted that irradiating the samples leads to nutrient loss, and similar observations were noted in our analyses (Figure 1). Since the diet has been reformulated to account for these losses, all the nutrients analyzed post-irradiation were above the NRC minimum requirements.

We analyzed primary and secondary lipid oxidation in the NuRFBs by measuring peroxide and p-anisidine levels and used these measurements to calculate TOTOX values. Food items for human consumption are considered fresh when peroxide levels are below 20 mEq/kg [38]. While there are no such guidelines for rodent food, the peroxide values in the NuRFBs were consistently significantly below the recommended 20 mEq/kg limit for human food, thus suggesting no dependence on time or storage temperature. p-Anisidine values in the NuRFBs remained stable in all groups and conditions except for an increasing trend at 6 months (both storage conditions) and a significant increase at the 27-month time point under refrigerated conditions. Higher p-anisidine values are reliable indicators of oxidative rancidity and lower storage stability [39]. Our results indicate that the NuRFBs are oxidatively stable and palatable for rodents at ambient conditions for 6 months, as well as at both refrigerated and refrigerated + ambient for up to 18 months, when kept in sterile closed conditions.

Contrary to our results, a previous study on NuRFB stability [12] documented the presence of oxidative rancidity in the NuRFBs stored at 26 °C (ambient temperature) at 6 months and oxidative stability of NuRFBs stored at 4 °C (refrigerated) for up to 12 months. They also reported significant losses in vitamin E, vitamin A, and thiamine in NuRFBs stored at 26 °C (ambient temperature) for 12 months, although they were well within the NRC recommendations. This study by Sun et al. (2012) concluded that NuRFBs stored at 4 °C for up to 12 months were suitable for rodent consumption, while those stored at 26 °C had a shorter shelf life of up to 6 months [12]. The disparity in the results was potentially due to distinct experimental designs and unaccounted variables, which varied from actual storage practices. In our study, a newly opened vacuum-packaged NuRFB sample (n = 3) was identically prepared for nutrient analysis for each condition at each time point. Meanwhile, Sun et al. (2012) used a single NuRFB sample (n = 1) that was stored at either 4 °C or 26 °C, and sampling was performed on one single bar across all time points in triplicate [12]. Sampling from the same opened NuRFB exposes the food bar to air, making it susceptible to oxidation and nutrient losses, especially at ambient temperatures. This is likely a major contributing factor to the observed accelerated lipid oxidation at ambient temperature in the Sun et al. (2012) study. This storage and usage practice does not align with actual storage or experimental use conditions (unopened, refrigerated until launch, and protected from light), and it is negligible to associate the results of the 2012 study from modern spaceflight food procedures.

In summary, no experimentally significant nutritional changes in the food bars over time were observed when stored in refrigerated, sealed conditions. Refrigeration is a vital component of long-term food bar storage, but the food bars are not severely affected at ambient temperature for 6 months and under combination of refrigerated and nominal ambient temperature conditions for up to 18 months. These data reflect the benefits of refrigerated storage yet show that there is room for some flexibility in logistical planning when preparing these food bars for ground and ISS studies with appropriate stability. With animal welfare as a primary concern in these missions, this study confirms that the vitamin content meets animal research norms for the mice and that the lipid oxidation indices are relatively stable for at least the duration of current experiments. Currently, food bars have an expiration date of 2 years post-extrusion, when they are stored at 4 °C. It was notable that this study did not show any growth of mold or yeast for the entire duration, suggesting that the current paradigm of microbial growth prevention (e.g., vacuum sealing, sorbating, and refrigeration) is sufficient to inhibit the growth of shelf-life-altering microorganisms.

Considering the complex nature of NASA flight projects and the lengthy production and labor-intensive process of the manufacture food bars, this is a critical study to document the changes in nutrient levels and lipid oxidation over an extended storage time and under varying storage temperatures. This study demonstrates that the current NuRFB production and handling processes produce food that meets animal research industry norms, as well as NRC guidelines.

## 5. Conclusions

The ISS environment has multiple variables, including increased radiation, reduced air convection, and altered humidity among others, all of which have the potential to impact the nutritional content of the food bars [1,5,40]. The evaluation of nutrient levels in the food bars from the RR5 mission revealed a reduction in retinol, vitamin B12, thiamine, vitamin B6, and vitamin D3 in ISS bars when compared to the ground controls. However, the levels of these vitamins were well within the NRC recommendations, suggesting no meaningful loss of nutrients in the ISS food bars over the short time course but that GCR may affect nutrient levels during longer missions or at increased radiation levels. Although the small sample size, dictated by the constraints of the spaceflight mission, is a limitation of this study, these preliminary findings offer valuable insights that can be further validated in future studies with larger sample sizes. In the ground-based shelf life test, some of the nutrients analyzed in certain groups showed statistically significant changes from their respective baseline, but these changes remained well still within the NRC recommendations. One of the striking observations was the low stability of thiamine at the ambient and refrigerated + ambient conditions, suggesting that refrigeration is likely required to maintain high thiamine levels. This is in agreement with other foods tested for spaceflight use [41]. Furthermore, riboflavin was stable up to 6 months at ambient and 18 months at the refrigerated + ambient conditions. Vitamin A and E levels were relatively stable in the NuRFBs under the tested conditions. We observed minor alterations in the vitamin D levels in the NuRFBs with slightly better stability under the refrigerated conditions. The changes observed in water-soluble and fat-soluble vitamins in all of the tested storage conditions exceeded the NRC minimums, thus suggesting long-term nutritional stability of the NuRFBs at the different combinations of storage conditions tested in this study. Due to the high risk of mold that comes from the high moisture content and water activity of these bars, which can cause mission failure, determining the storage and shelf life of rodent food bars has been critical to ensure animals are fed clean and palatable food during NASA missions. One crucial modification would be to reformulate these bars with a low moisture level (as is found in commercial rodent chow) to reduce microbial load and possibly increase shelf life for longer duration missions beyond low earth orbit planned for the near future.

## Figures and Tables

**Table 1:** NuRFB Dry diet formulation.

Ingredient	Quantity (g/kg)
Casein	100
DL-Methionine	3
Wheat Gluten	120
Wheat Flour, Durum 2nd clear	225
Corn Starch	198.889
Corn Syrup	100
Sucrose	100
Soybean Oil	40
Cellulose	50
Mineral Mix, AIN-93G-MX	35
Calcium Carbonate	5
Vitamin Mix, AIN-93-VX	20
Choline Bitartrate	2.5
Vitamin B12	0.23
Thiamin (81%)	0.36
Folic Acid	0.012
Vitamin K1, phylloquinone	0.001
TBHQ, antioxidant	0.008

**Table 2:** Experimental design (AT = ambient temperature).

Sample Group	Time Under Refrigeration (Months)	Time at Ambient Temperature (Months)	Rationale/Objective
Baseline	0	0	Baseline value before storage
1	0	6	Bag prepared using nitrogen gas
1A	0	6	Bag prepared using ethylene oxide
2	3	3	Flight scenario replication
3	3	6	Flight scenario replication
4A	6	0	Extended storage at 4 °C with transfer to AT
4B	6	6	
5A	12	0	Longer term storage at 4 °C with transfer to AT
5B	12	6	
6	27	0	Refrigerated control group (4 °C)

**Table 3:** Markers assessed in the study. List of vitamins assessed with the NRC minimum requirements in parentheses, list of lipid oxidation markers, and microbiology markers assessed.

Vitamins	Lipid Oxidation Markers	Microbiology
Vitamin A as Retinol (>2.4 IU/g)	Peroxide value	Mold
Vitamin D_3_ (>1 IU/g)	p-Anisidine	Yeast
Vitamin E (>0.0320 IU/g)	TOTOX	Total Aerobic Bacteria
Thiamine (>0.50 mg/100 mg)		Total Anerobic Bacteria
Riboflavin (>7 mg/g)		

## Data Availability

The original contributions presented in this study are included in the article/Supplementary Materials. Further inquiries can be directed to the corresponding author.

## Supplementary Materials

The following supporting information can be downloaded at https://www.mdpi.com/article/10.3390/foods13244093/s1. Figure S1: Fat-soluble vitamin levels measured across different treatment groups. Subtle non-significant fluctuations were noted in vitamin A (A) and vitamin E (B) levels compared to the baseline B. (C) Significant decrease in vitamin D3 levels were observed in the 5B group (12 months refrigeration followed by 6 months at ambient temperature) compared to the baseline-B. The dotted line corresponds to the NRC minimum requirements for the respective vit-amins. Figure S2. Lipid oxidation in NuRFBs. (A) A decreasing trend was observed in the peroxide levels across groups compared to the baseline, with significant decreases in groups 4B (6 months refrigeration followed by 6 months at ambient temperature, *p* = 0.0047) and 5A (12-month refrigeration, *p* = 0.0047) compared to the baseline-B. (B) Non-significant fluctuations in p-anisidine values were noted across all groups. (C) Fluctuations in TOTOX values were observed with a significant decrease in group 5A (12-month refrigeration, *p* = 0.0363) compared to the baseline-B. (D) A significant decrease in peroxide levels was observed at the 6-month time point in ambient (*p* = 0.0238) and at the 12-month time point in refrigerated (*p* = 0.0062) and refrigerated + ambient (*p* = 0.0074) storage conditions. (E) Significant increases in p-anisidine values were noted at the 27-month (*p* = 0.05) time point in refrigerated storage conditions. (F) Significant decreases in TOTOX values were observed at the 6-month time point in ambient (*p* = 0.0476) and at the 12-month point in refrigerated + ambient (*p* = 0.0423) storage conditions. Figure S3: Yeast and mold counts in NuRFBs. No significant changes were observed in mold (A,C) and yeast (B,D) counts across the groups and over time. Figure S4: Microbial plate counts measured across different treatment groups. No significant changes were ob-served in aerobic (A) and anaerobic (B) plate counts across the groups.

## References

[1] Afshinnekoo E., Scott R.T., MacKay M.J., Pariset E., Cekanaviciute E., Barker R., Gilroy S., Hassane D., Smith S.M., Zwart S.R. (2020). Fundamental Biological Features of Spaceflight: Advancing the Field to Enable Deep-Space Exploration. Cell.

[2] Garrett-Bakelman F.E., Darshi M., Green S.J., Gur R.C., Lin L., Macias B.R., McKenna M.J., Meydan C., Mishra T., Nasrini J. (2019). The NASA Twins Study: A multidimensional analysis of a year-long human spaceflight. Science.

[3] Mhatre S.D., Iyer J., Petereit J., Dolling-Boreham R.M., Tyryshkina A., Paul A.M., Gilbert R., Jensen M., Woolsey R.J., Anand S. (2022). Artificial gravity partially protects space-induced neurological deficits in Drosophila melanogaster. Cell Rep..

[4] Crucian B.E., Chouker A., Simpson R.J., Mehta S., Marshall G., Smith S.M., Zwart S.R., Heer M., Ponomarev S., Whitmire A. (2018). Immune System Dysregulation During Spaceflight: Potential Countermeasures for Deep Space Exploration Missions. Front. Immunol..

[5] Tou J., Grindeland R., Barrett J., Dalton B., Mandel A., Wade C. (2003). Evaluation of NASA Foodbars as a standard diet for use in Short-Term rodent space flight studies. Nutrition.

[6] Sun G.-S., Tou J.C., Yu D., Girten B.E., Cohen J. (2014). The past, present, and future of National Aeronautics and Space Administration spaceflight diet in support of microgravity rodent experiments. Nutrition.

[7] Franklin C.L., Ericsson A.C. (2017). Microbiota and reproducibility of rodent models. Lab Anim..

[8] Tuck C.J., De Palma G., Takami K., Brant B., Caminero A., Reed D.E., Muir J.G., Gibson P.R., Winterborn A., Verdu E.F. (2020). Nutritional profile of rodent diets impacts experimental reproducibility in microbiome preclinical research. Sci. Rep..

[9] A Pellizzon M., Ricci M.R. (2020). Choice of Laboratory Rodent Diet May Confound Data Interpretation and Reproducibility. Curr. Dev. Nutr..

[10] National Research Council (US) (1995). Subcommitee on Laboratory Animal Nutrition. Nutrient Requirements of Laboratory Animals.

[11] Reeves P.G., Nielsen F.H., Fahey G.C. (1993). AIN-93 Purified Diets for Laboratory Rodents: Final Report of the American Institute of Nutrition Ad Hoc Writing Committee on the Reformulation of the AIN-76A Rodent Diet. J. Nutr..

[12] Sun G.-S., Tou J.C., Reiss-Bubenheim D.A., Hill E.L., Liittschwager K.W., Girten B.E., Pena-Yewkukhiw E. (2012). Oxidative and nutrient stability of a standard rodent spaceflight diet during long-term storage. Lab Anim..

[13] Tao L. (2015). Oxidation of Polyunsaturated Fatty Acids and its Impact on Food Quality and Human Health. Adv. Food Technol. Nutr. Sci. Open J..

[14] AOAC (2005). Vitamin A—Association of Official Analytical Chemists. Official Methods Anal. AOAC International 18th Ed. Method 992.06.

[15] AOAC (2005). Vitamin A—Association of Official Analytical Chemists. Official Methods Anal. AOAC International 18th Ed. Method 992.04.

[16] Cort W.M., Vicente T.S., Waysek E.H., Williams B.D. (1983). Vitamin E content of feedstuffs determined by high-performance liquid chromatographic fluorescence. J. Agric. Food Chem..

[17] Speek A.J., Schrijver J., Schreurs W.H.P. (2006). Vitamin E Composition of Some Seed Oils as Determined by High-Performance Liquid Chromatography with Fluorometric Detection. J. Food Sci..

[18] McMurray C.H., Blanchflower W.J., A Rice D. (1980). Influence of Extraction Techniques on Determination of α-Tocopherol in Animal Feedstuffs. J. AOAC Int..

[19] AOAC (2005). Vitamin D3—Association of Official Analytical Chemists. Official Methods Anal. AOAC International 18th Ed. Method 2011.11.

[20] AOAC (2005). Riboflavin—Association of Official Analytical Chemists. Official Methods Anal. AOAC International 18th Ed. Method 940.33.

[21] AOAC (2005). Riboflavin—Association of Official Analytical Chemists. Official Methods Anal. AOAC International 18th Ed. Method 960.46.

[22] AOAC (2005). Thiamin—Association of Official Analytical Chemists. Official Methods Anal. AOAC International 18th Ed. Method 942.33.

[23] AOAC (2005). Thiamin—Association of Official Analytical Chemists. Official Methods Anal. AOAC International 18th Ed. Method 953.17.

[24] AOAC (2005). Thiamin—Association of Official Analytical Chemists. Official Methods Anal. AOAC International 18th Ed. Method 957.17.

[25] AOAC (2005). Peroxide—Association of Official Analytical Chemists. Official Methods Anal. AOAC International 18th Ed. Method 965.33.

[26] United States Pharmacopeia (USP) (2013). 36th Rev, ‘Peroxide Value’. Fats & Fixed Oils.

[27] AOAC (2005). p-Anisidine—Association of Official Analytical Chemists. Official Methods Anal. AOAC International 18th Ed. Method Cd 18-90.

[28] United States Pharmacopeia (USP) (2015). 38th Rev, “p-Anisidine Value”. Fats & Fixed Oils.

[29] AOAC (2005). Moisture—Association of Official Analytical Chemists. Official Methods Anal. AOAC International 18th Ed. Method 925.09.

[30] AOAC (2005). Moisture—Association of Official Analytical Chemists. Official Methods Anal. AOAC International 18th Ed. Method 926.08.

[31] AOAC (2005). Aerobic bacteria—Association of Official Analytical Chemists. Official Methods Anal. AOAC International 18th Ed. Method 966.23.

[32] Maturin L., Peeler J. (2001). Chapter 3. Aerobic Plate Count. Food and Drug Administration (FDA), Bacteriological Analytical Manual Online.

[33] Beetner G., Tsao T., Frey A., Harper J. (1974). Degradation of Thiamine and Riboflavin during extrusion processing. J. Food Sci..

[34] Connell J. (1975). Control of Fish Quality.

[35] Tang H., Rising H.H., Majji M., Brown R.D. (2021). Long-Term Space Nutrition: A Scoping Review. Nutrients.

[36] Mironeasa S., Coţovanu I., Mironeasa C., Ungureanu-Iuga M. (2023). A Review of the Changes Produced by Extrusion Cooking on the Bioactive Compounds from Vegetal Sources. Antioxidants.

[37] Riaz M.N., Asif M., Ali R. (2009). Stability of Vitamins during Extrusion. Crit. Rev. Food Sci. Nutr..

[38] Kong F., Singh R.P. (2011). Advances in instrumental methods to determine food quality deterioration. Food and Beverage Stability and Shelf Life.

[39] Irwin J.W., Hedges N. (2004). Measuring lipid oxidation. Understanding and Measuring the Shelf-Life of Food.

[40] Salehi Pourbavarsad M., Jalili Jalalieh Behnaz Owuor J., Jackson W.A., Muirhead D. Humidity Condensate Stabilization Using an Engineered Biologically Active Storage Tank. Proceedings of the 50th International Conference on Environmental System.

[41] Cooper M., Perchonok M., Douglas G.L. (2017). Initial assessment of the nutritional quality of the space food system over three years of ambient storage. NPJ Microgravity.

