# Supplementary material for: Nutrient Stability in NASA Spaceflight Experiment Rodent Food Bars
Source: Foods. 2024 Dec 18;13(24):4093. doi: 10.3390/foods13244093 (PMC11675554; doi:10.3390/foods13244093)
Supplement: Supplementary file 1 [file foods-13-04093-s001.zip › foods-3245240-supplementary.pdf]

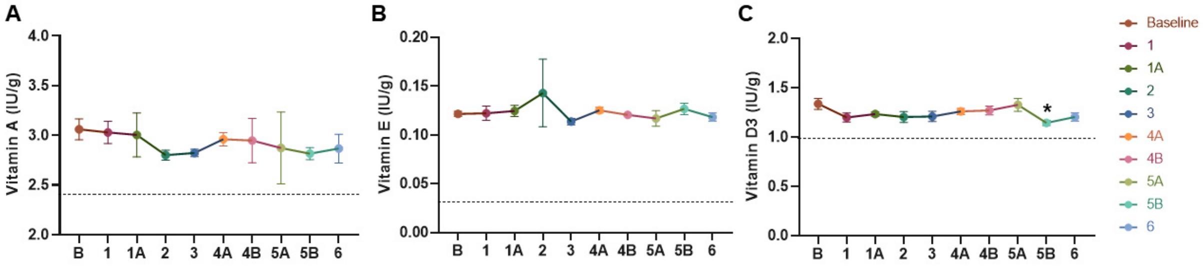

Figure S1: Fat-soluble vitamin levels measured across different treatment groups. Subtle non-significant fluctuations were noted in Vitamin A (A) and Vitamin E (B) levels compared to the baseline B. **(C)** Significant decrease in Vitamin D3 levels were observed in the 5B group (12 months refrigeration followed by 6 months at ambient temperature – Black *) compared to the baseline-B. The dotted line corresponds to the NRC minimum requirements for the respective vitamins.

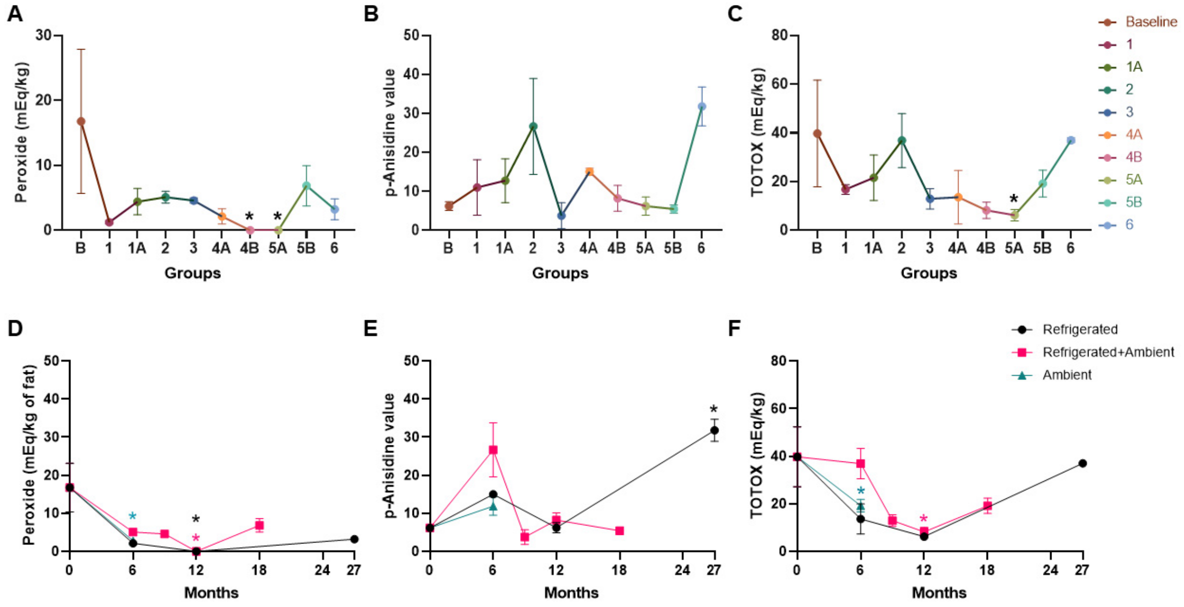

Figure S2. Lipid oxidation in NuRFBs. (A) A decreasing trend was observed in the peroxide levels across groups compared to the baseline, with significant decreases in groups 4B (6 months refrigeration followed by 6 months at ambient temperature, p=0.0047, Black *) and 5A (12-month refrigeration, p=0.0047, Black *) compared to the baseline-B. **(B)** Non-significant fluctuations in p-anisidine values were noted across all groups. **(C)** Fluctuations in TOTOX values were observed with a significant decrease in group 5A (12-month refrigeration, p=0.0363, Black *) compared to the baseline-B. **(D)** A significant decrease in peroxide levels was observed at the 6-month time point in ambient (p=0.0238, Teal *), and at the 12-month time point in refrigerated (p=0.0062, Black *) and refrigerated+ambient (p=0.0074, Pink *) storage conditions. **(E)** Significant increases in p-anisidine values were noted at the 27-month (p=0.05, – Black *) time point in refrigerated storage conditions. **(F)** Significant decreases in TOTOX values were observed at the 6-month time point in ambient (p=0.0476, Teal), and at the 12-month point in refrigerated+ambient (p=0.0423, Pink *) storage conditions.

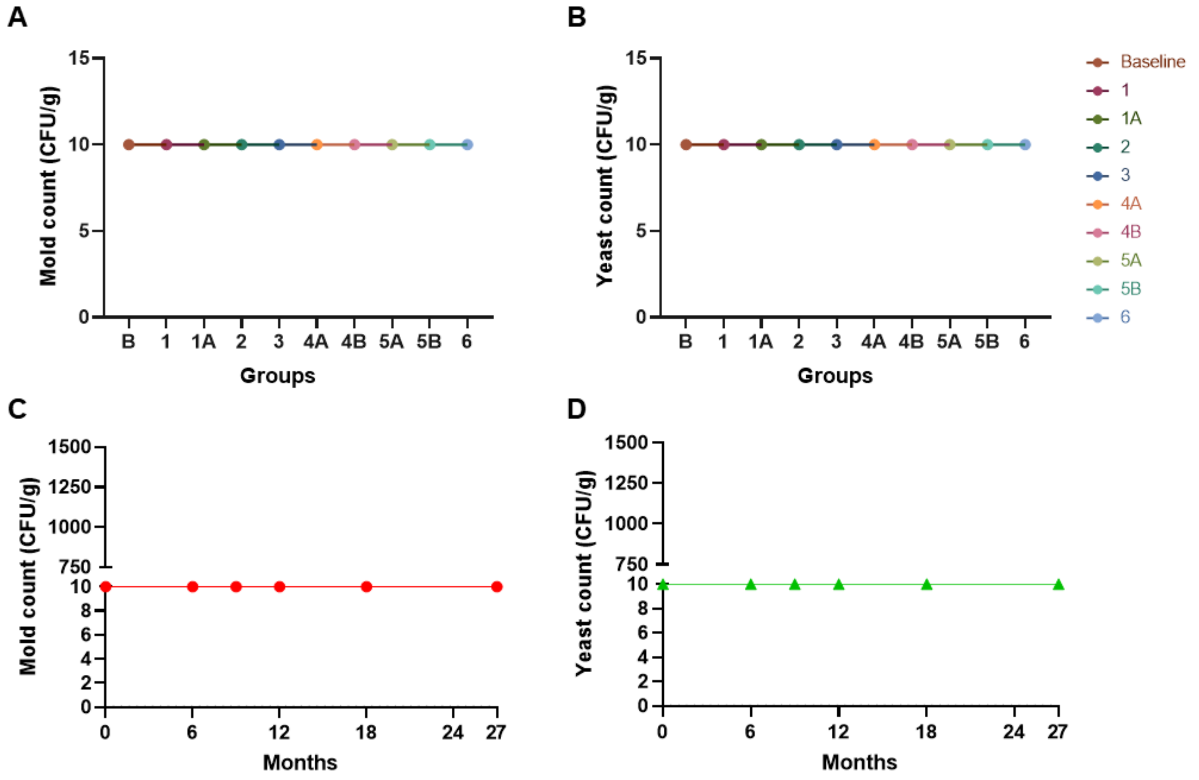

Figure S3: **Yeast and mold counts in NuRFBs.** No significant changes were observed in mold (**A, C**) and yeast (**B, D)** counts across the groups and over time.

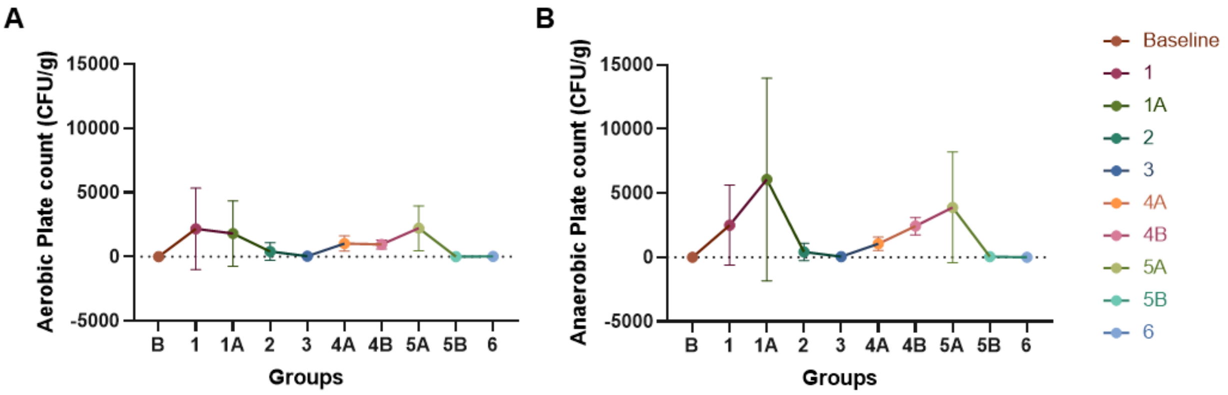

Figure S4: Microbial plate counts measured across different treatment groups. No significant changes were observed in aerobic (**A**) and anaerobic (**B)** plate counts across the groups.
